# Dynamics of Cell Death Due to Electroporation Using Different Pulse Parameters as Revealed by Different Viability Assays

**DOI:** 10.1007/s10439-023-03309-8

**Published:** 2023-09-13

**Authors:** Wencheng Peng, Tamara Polajžer, Chenguo Yao, Damijan Miklavčič

**Affiliations:** 1 The State Key Laboratory of Power Transmission Equipment and System Security and New Technology, School of Electrical Engineering, Chongqing University, Chongqing, 400044 China; 2 Faculty of Electrical Engineering, University of Ljubljana, Tržaška 25, 1000 Ljubljana, Slovenia

**Keywords:** Electroporation, Pulsed field ablation, Cell death dynamics, Pulse parameters, Viability assays

## Abstract

The mechanisms of cell death due to electroporation are still not well understood. Recent studies suggest that cell death due to electroporation is not an immediate all-or-nothing response but rather a dynamic process that occurs over a prolonged period of time. To investigate whether the dynamics of cell death depends on the pulse parameters or cell lines, we exposed different cell lines to different pulses [monopolar millisecond, microsecond, nanosecond, and high-frequency bipolar (HFIRE)] and then assessed viability at different times using different viability assays. The dynamics of cell death was observed by changes in metabolic activity and membrane integrity. In addition, regardless of pulse or cell line, the dynamics of cell death was observed only at high electroporation intensities, i.e., high pulse amplitudes and/or pulse number. Considering the dynamics of cell death, the clonogenic assay should remain the preferred viability assay for assessing viability after electroporation.

**Supplementary Information:**

The online version contains supplementary material available at 10.1007/s10439-023-03309-8.

## Introduction

The exposure of cell to high pulsed electric fields or so-called electroporation causes structural and chemical changes in the plasma membrane [11], therefore damaging the cell membrane. The damage triggers repair mechanisms, which include patching, clogging of the pores, as well as replacing damaged lipids and proteins by endo- and exocytosis in order to reestablish homeostasis and cell functioning [1, 17]. If repair mechanisms are successful, we consider electroporation to be reversible (RE). However, sometimes the damage to the cell caused by electroporation is too severe and fatal because the cell cannot repair the damage or restore metabolism, so the cell will die. This is considered to be irreversible electroporation (IRE). Reversible electroporation in the field of medicine is mostly used for cancer treatment or gene therapy, where transient damages (leading to transient changes in plasma permeability) allow therapeutic molecules (specific cancer drugs or nucleic acids) to enter the target cell [5, 14, 30]. In ablation where the main goal of the therapy is inducing cell death [2], IRE is used for ablation of soft tumors in liver [12], prostate [6], pancreas [28], and kidney [19]. IRE is rapidly developed and is being accepted also in the field of cardiac ablation (as pulsed field ablation) for the treatment of cardiac arrhythmias, such as atrial fibrillation [24].

The effectiveness of electroporation depends on pulse parameters such as duration (milliseconds, microseconds, nanoseconds), number of pulses, frequency, strength of the electric field, and pulse type (monopolar or bipolar pulses) [22]. The same parameters are believed to affect cell death as well [17]. Cell death caused by electroporation has been studied since the beginning of electroporation studies. However, the mechanisms of cell death and signaling pathways after electroporation are still not well understood [17].

For a cell to die, it must cross the point-of-no-return. Cell injury must be severe to be irreparable and result in cell death. During the dying process, different events can take place in the cell simultaneously or successively: plasma membrane has lost its integrity, the activity of intracellular enzymes decreases, the ATP content decreases, which affects the cellular energy capacity, and genetic material is fragmented [15]. While some of the changes in the dying cell can be observed using a viability assays, detecting the cell death type and pathways requires gene and protein analysis [9]. Viability assays have been developed to determine exactly how a stressor (chemical or physical) can modulate cell death. These assays provide information about the physical and physiological health of cells after their treatment in vitro. In addition, viability assays are the basic methods to investigate cell death/viability in relation to plasma membrane integrity in electroporation research field [32].

Recent studies show that differences in cell death can be observed at different time points after electroporation, using simple viability assay. It seems that cell death in electroporation-based treatment is not an immediate all-or-nothing response but results in the dynamics of cell death, making cell death more complex than originally thought. It was suggested that cell death due to electroporation is immediate and delayed [13, 16, 31]. First, a portion of the cells cannot repair their membrane integrity after electroporation, which leads to accidental cell death—necrosis. Second, the rest of the cells manage to restore their membrane integrity and later die by regulated cell death (apoptosis, necroptosis, and pyroptosis) [13]. It seems that cell survival depends first on cell's ability to repair (membrane) damage and second on cell's ability to restore homeostasis. Moreover, studies show that dynamics of cell death is also related to other pulse parameters, like the strength of electric field [13, 16, 31]. Dynamics of cell death was recently confirmed also with the shortest nanosecond pulses [33].

Furthermore, dynamics of cell death after electroporation was shown as well through gene expression analysis [26]. Different signaling pathways related to three biological functions of the cell (cell injury, cell death, and inflammation) were triggered at different time points after electroporation. Immediately after electroporation, analysis showed upregulation of genes associated with cell injury. As early as 2 h after electroporation, these genes were downregulated, and the upregulation shifted to genes associated with apoptosis. Apoptosis genes were downregulated within the first 8 h and were no longer present within 24 h. 24 h after electroporation, there was an increase in gene expression related to inflammation, repair, regeneration, pyroptosis, and necrosis (immunogenic responses). This indicates that cell death due to electroporation is a dynamic process that extends over a prolonged period of time.

Recent review on cell death in response to electroporation revealed that the lack of experimental data hinders the understanding of cell death in electroporation-based therapies. Currently obtained results are quite often contradictory [17]. However, since recent studies suggest that cell death in electroporation is not an immediate all-or-nothing response but may be a dynamic process that occurs over time [13, 31, 33], dynamics of cell death could explain why different studies have produced conflicting results. The aim of our study was to further explore the dynamics of cell death following electroporation. To assess the dynamics of cell death, different viability assays were performed at different times after electroporation. Hopefully, this will result in the determination of the best time and assay for cell death evaluation. Since it is known that efficacy of electroporation depends on pulse parameters, different pulses, different durations, types, and amplitudes were investigated. We exposed cells to millisecond, microsecond, and nanosecond monopolar pulses and high-frequency bipolar (HFIRE) pulses. In addition, cell lines of different origin were used to investigate if dynamics of cell death is cell type dependent. Understanding the dynamics of cell death and how this correlates with different viability assays over a timeline will hopefully the improve overall understanding of cell death in vitro and help to better understand and develop the electroporation-based therapies.

## Materials and Methods

### Cells

Chinese hamster ovary cells (CHO), mouse melanoma cells (B16F1), and rat heart myoblast cells (H9c2) were purchased from European Collection of Authenticated Cell Cultures. CHO cells were grown in HAM F-12 growth medium (PAA, Austria), and B16F1 and H9c2 were grown in Dulbecco’s Modified Eagle’s Medium (DMEM) growth medium (Sigma-Aldrich, USA). All media were supplemented with 10% fetal bovine serum (FBS, Sigma-Aldrich, USA), L-glutamine (0.5% for CHO, 1% for B16F1, 2% for H9c2) (StemCell, Canada), penicillin/streptomycin (PAA, Austria), and 0.1% gentamycin (Sigma-Aldrich, USA). Cells were grown in an incubator at 37 °C with controlled atmosphere (CHO and B16F1 at 5% CO_2_, H9c2 at 10% CO_2_) until 70–80% confluency was reached. Afterward, the growth medium was removed and the trypsin-EDTA (PAA, Austria) was used to detach cells. Afterward, fresh medium was added to inactivate trypsin. Cell suspension was then centrifuged at 180 g for 5 minutes, supernatant was removed and the cells were resuspended to a desired cell density in their own fresh growth medium (with all the supplements), which was used as an electroporation buffer. Cell density of 3 × 10^6^ cells/ml was used for sample size of 150 µl, used for millisecond (ms), microsecond (µs), HFIRE and 200 nanosecond (ns) pulses. For 4 ns pulses, sample size had to be adjusted to 60 µl due to impedance matching between generator and biological load (cuvette). In this case, cell density was increased to 5 × 10^6^ cells/ml to obtain enough cells for analysis. Samples were then transferred to 2 mm aluminum cuvettes (VWR International, USA) and pulses delivered.

### Pulse Delivery

Cells were exposed to different electroporation pulses, as described in Table 1. Millisecond (ms) pulses were applied with laboratory prototype pulse generator, as previously described in [34]. Microsecond (µs) and HFIRE (2 µs-positive pulse–2 µs interphase delay–2 µs-negative pulse–2 µs interpulse delay, (2–2–2–2 pattern)) pulses were applied with high-frequency prototype pulse generator L-POR V0.1 (mPOR, Slovenia), as previously described in [21]. All pulses were monitored by a high-voltage differential probe HVD3605A (Teledyne LeCroy, USA), current probe CP031 (Teledyne LeCroy, USA), and HDO6000 High-Definition oscilloscope (Teledyne LeCroy, USA). Electroporator CellFX System electroporator (Pulse Biosciences, USA) was used for the delivery of 200 ns pulses. Voltage and current were measured by Pearson current monitor model 2877 (Pearson Electronics, USA), Pearson current monitor model 2878 (Pearson Electronics, USA), respectively, and oscilloscope WaveSurfer 3024Z, 200 MHz (Teledyne LeCroy, USA). Electroporator FPG20-1NM4 (FID Technology, Germany) was used to deliver 4 ns pulses, which were measured by high-voltage coupler (CPF 30L50-B500-D40, 50 Ohm, DC-500 MHz, FID GmbH, Germany), with division ration 57.8 dB (1:780), placed between two high-voltage cables FC26, FID GmbH, Germany). High-voltage coupler was connected with SMD cable in − 20 dB attenuator (1:10, 50 Ohm, DC-1 GHz, Telegärtner, Germany) to oscilloscope WaveSurfer 3024Z, 200 MHz (Teledyne LeCroy, USA). Same experimental setting was used in our recent study, where we present as well measured current and volage [20]. Based on permeability and survival curves presented in our recent study, electric field and pulse number resulting in 90–10% survival (based on MTS after 24 h were chosen) were determined, to investigate if cell death dynamics is pulse intensity dependent.

**Table 1:** Parameters of electroporation pulses

Pulse duration/type	Fixed pulse parameters	Variable parameter	Electroporation intensity
5 ms	8 pulses, 1 Hz	Voltage (V): 0, 100, 150, 175, 200, 250	Electric field (V/cm): 0, 500, 750, 875, 1000, 1250
100 μs	8 pulses, 1 Hz	Voltage (V): 0, 100, 200, 300, 400, 500	Electric field (V/cm): 0, 500, 1000, 1500, 2000, 2500
200 ns	100 pulse, 10 Hz	Voltage (V): 0, 1000, 2000, 3000, 4000, 5000	Electric field (V/cm): 0, 5000, 10000, 15000, 20000, 25000
4 ns	500 Hz, 12 kV	Number of pulses: 0, 1000, 2000, 3000, 4000, 5000	Number of pulses: 0, 1000, 2000, 3000, 4000, 5000
2–2–2–2 µs (HFIRE)	32 pulses in a burst, 100 bursts with 1 Hz	Voltage (V): 0, 100, 200, 300, 400, 500	Electric field (V/cm): 0, 500, 1000, 1500, 2000, 2500

### Viability Assays

#### Clonogenic Assay

After pulse application, samples were diluted in fresh growth media. 100 cells were plated in 6-well plate (TPP, Switzerland) with 2.5 ml of growth media and returned to the incubator for 7 days for all cell lines. Then medium was removed and the cells were stained with 1 ml of 0.2% crystal violet/methanol solution for 15 minutes. Afterward, the cells were washed with water and colonies counted. The survival was calculated by normalizing the number of colonies of the samples to the number of colonies of the sham control.

#### Metabolic Assay

MTS assay (CellTiter 96® AQueous One Solution Cell Proliferation Assay, Promega, USA) was used as a metabolic assay to evaluate the viability after electroporation. After pulse treatment, cells were diluted in fresh growth medium and transferred to 96-well plate (TPP, Switzerland). 2.2 × 10^4^ cells were transferred into the 96-well plate for 1, 2, 4, and 8 h, 1.8 × 10^4^ cells for 12 h, 1.1 × 10^4^ cells for 24 h, 5.5 × 10^3^ cells for 48 h, and 2.25 × 10^3^ cells for 72 h. Appropriate amount of fresh growth media was added to samples, so that final volume in 96-well plate was 100 μl. To avoid evaporation, we added saline solution in the outer wells of 96-well plate. Cells were then returned to the incubator until analysis. Analysis was performed according to the manufacturer instruction. 20 μl of MTS solution was added to each well and incubated in the incubator for 1 h. Afterward, the absorbance of reduced MTS solution was detected with a spectrofluorometer (Tecan Infinite M200, Tecan, Austria) at 490 nm. The percentage of viable cells was calculated by normalizing the absorbance of the samples to the absorbance of the sham control for each time point. Furthermore, if evaporation or any color changing would occur in 72 h, this would occur in all samples, including control. Since the results were normalized to the control (each timepoint had its own control), normalization nullified these changes.

##### Membrane Integrity Assay—Propidium Iodide

After pulse application, cells were diluted in fresh growth media. For each time point (1, 2, 4, 8, 12, 24, 48, and 72 h) in membrane integrity assay, 10^4^ cells were transferred to 1 ml of fresh media in 12-well plate (TPP, Switzerland). Cells were then returned to the incubator until analysis. For analysis, cells were harvested (attached and unattached) and centrifuged at 500 g for 1 min. Cell pellet was then resuspended in 100 μl of fresh growth medium. PI was added to final concentration of 100 µg/ml and cells were incubated at room temperature for five minutes. Membrane integrity was analyzed by flow cytometer (Attune NxT; Life Technologies, USA), using 488 nm blue laser and 574/26 nm band-pass filter. The number of analyzed events was set to 10,000. Attune Nxt software was used for data interpretation. Dot-plot of forward-scatter and side-scatter was used for elimination of debris and clusters from analysis, so only single cells were taken into further analysis. Based on the signal of sham control (0 V), gates for live (lowest fluorescence—negative PI signal) and dead cells (highest fluorescence- very strong PI signal) were set. Between the gate of live and dead cell, another gate was identified. Through this gate, we could observe the increase in fluorescence due to the uptake of the cell PI as a result of the damaged membrane of the living cell (Fig. 4). This PI gating strategy previously exploited by ([8, p. 198]; [27]) to distinguish between nonelectroporated live cells, electroporated live cells with transiently permeable membrane and cells with permanent membrane damage. For the detection of changes in membrane permeability, over the 72 h same gating strategy was taken, but for different interpretation of the results. In our case gating was dividing cells to live cells with intact plasma membrane, live cells with some damages to the membrane which allows some PI molecules to enter the cell (probably cells that are dying) and dead cells with complete permeability to PI.

### Statistical Analysis

All experiments were repeated at least three times. The results are shown as mean ± SD. Statistical analysis was performed using SigmaPlot 11.0 (Systat Software, USA). Statistically significant differences (*p < 0.05) were determined by one-way ANOVA test and Holm-Sidak post hoc test.

## Results

**Fig. 1:**
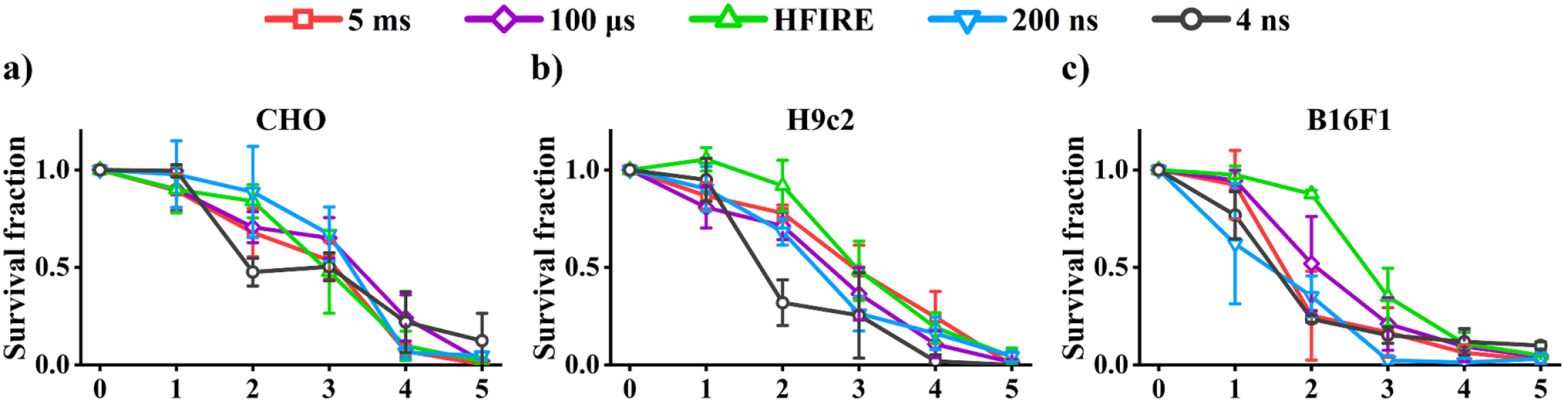
Clonogenic assay. Survival fraction is plotted against the intensities of electroporation (in amplitude for 5 ms, 100 µs, HFIRE, 200 ns and number of pulses for 4 ns). Amplitudes and pulse numbers are described in Table 1. **a** CHO; **b** H9c2 and **c** B16F1. 0–Sham control (0 V; 0 pulses) for 5 ms, 100 μs, HFIRE, 200 ns, and 4 ns; 1–100 V for 5 ms, 100 μs, and HFIRE, 1000 V for 200 ns, 1000 pulses for 4 ns; 2–150 V for 5 ms, 200 V for 100 μs and HFIRE, 2000 V for 200 ns, 2000 pulses for 4 ns; 3–175 V for 5 ms, 300 V for 100 μs and HFIRE, 3000 V for 200 ns, 3000 pulses for 4 ns; 4–200 V for 5 ms, 400 V for 100 μs and HFIRE, 4000 V for 200 ns, 4000 pulses for 4 ns; 5–250 V for 5 ms, 500 V for 100 μs and HFIRE, 5000 V for 200 ns, 5000 pulses for 4 ns

Clonogenic assay, known as the most reliable viability assay, was used to assess cell death after electroporation. Clonogenic results, assessed 7 days after treatment and as such provide ultimate results of IRE, show that all pulses used in this study can achieve IRE. As expected, results show that the efficacy of IRE depends on intensity of electroporation, i.e., voltage (electric field) or pulse number (for 4 ns pulses) (Fig. 1). The lowest intensity electroporation results in RE, with survival fraction > 0.8. Increase of electroporation intensity leads to lower survival fraction. Furthermore, sometimes even at the highest voltages, some cells survived and maintained their proliferative capacity, resulting in formation of a few (1–5) colonies.

**Fig. 2:**
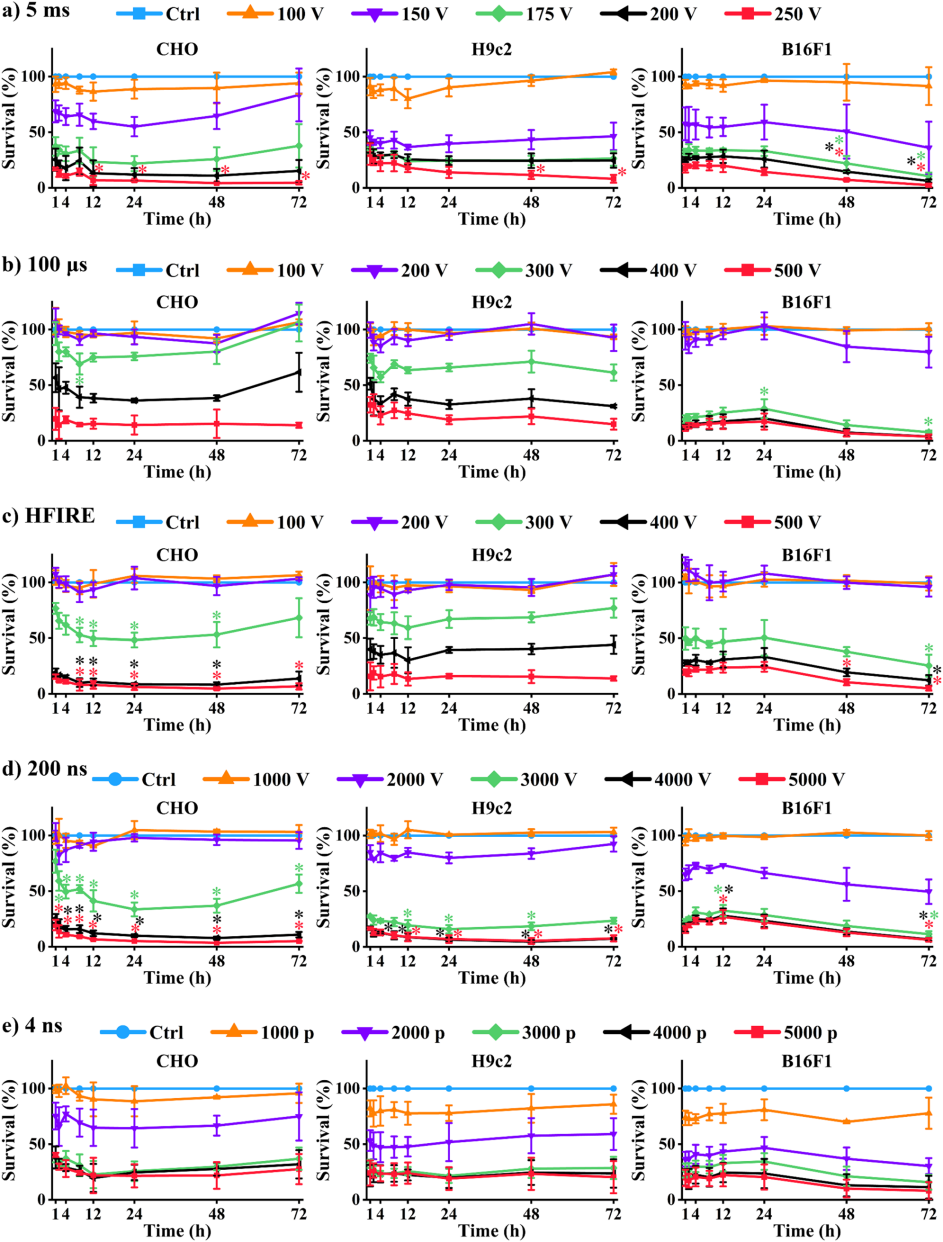
Dynamics of cell death detected using metabolic assay. Statistically significant differences between metabolic activity at 1 h after electroporation and metabolic activity at different time points after electroporation are shown. **a** 5 ms; **b** 100 µs; **c** HFIRE; **d** 200 ns; **e** 4 ns. For all the pulses left is CHO, middle H9c2, right B16F1

However, clonogenic assay does not offer insight into the dynamics of cell death. Therefore, additional viability assays were performed. Decreased metabolism of the cell population is considered a sign of cell death. Therefore, a metabolic assay was used to investigate if the dynamics of cell death is reflected in changes in metabolism after electroporation. Metabolic activity was assessed using MTS at 1, 2, 4, 8, 12, 24, 48, and 72 h after electroporation (Fig. 2). This timeline allowed us to distinguish between immediate and delayed changes in metabolic activity, thus changes in survival. Results obtained at 1 h after electroporation were considered immediate, while results at other time points were considered delayed. Therefore, metabolic activity at different time points was compared to metabolic activity at 1 h. In addition, pulses of different intensities (amplitudes/number or pulses) were used to determine if the dynamics of cell death is electroporation intensity dependent.

In all three cell lines, statistically significant differences (*p* < 0.05) in metabolism activity were never observed when pulses causing more than 0.8 survival fraction (cell viability) were used (Fig. 2). This indicates that the presence of dynamics of cell death depends on the intensity of electroporation. Using higher voltages and pulse number resulted in statistically significant differences between immediate and delayed metabolic activity. Nevertheless, these statistically significant differences varied between different time points, chosen voltage/pulse number, and even between the cell lines.

In CHO cells, 5 ms, 100 µs, HFIRE, and 200 ns pulses caused continuously decrease in metabolic activity/survival during the first 24 h. Nevertheless, statistically significant differences were not observed for all the data points and were most pronounced for HFIRE and 200 ns pulses. After 24 h, metabolic activity increased, assumingly due to cell proliferation, which results in more metabolically active population. H9c2 cells showed similar trend as CHO cells. However, in H9c2 cells, statistically significant differences in metabolic activity/survival were detected when 5 ms and 200 ns pulses were used. B16F1 responded to pulses somewhat differently, as within first 12 h, there was an increase or no change in metabolic activity. Metabolic activity in B16F1 was decreased at 48 or 72 h after electroporation, depending on the pulse length and pulse type. This decrease of metabolic activity was unexpected, as we assumed that due to proliferation capacities, and larger number of cell metabolic activity would be enhanced. For all three cell lines, there was no dynamics of cell death observed after electroporation with 4 ns pulses. Based on the obtained results, we can conclude that some dynamics of cell death can be observed with the metabolic assay, but the reasons for that need to be elucidated.

Cell death can also be assessed by testing membrane integrity, as disrupted membrane or damaged membrane is considered a sign of cell death. Therefore, we also used a membrane integrity assay to investigate if the dynamics of cell death is reflected in membrane integrity after electroporation. Since electroporation by itself causes increased permeability of the membrane, i.e., renders membrane transiently permeable, such viability assay in electroporation studies has low reliability, specialty soon after electroporation [32]. To separate transient damage caused by electroporation (transient pore formation) from actual damage in the cell membrane, internalization of propidium iodide (PI) was assessed at 1, 2, 4, 8, 12, 24, 48, and 72 h after electroporation. One-hour time point was used as immediate cell death, while the results obtained at other time points were considers as delayed cell death. This timeline allowed us to distinguish between immediate and delayed changes in membrane permeability, thus changes in survival. In addition, pulses of different intensities (voltages/number or pulses) were used to determine if the dynamics of cell death is electroporation intensity dependent.

**Fig. 3:**
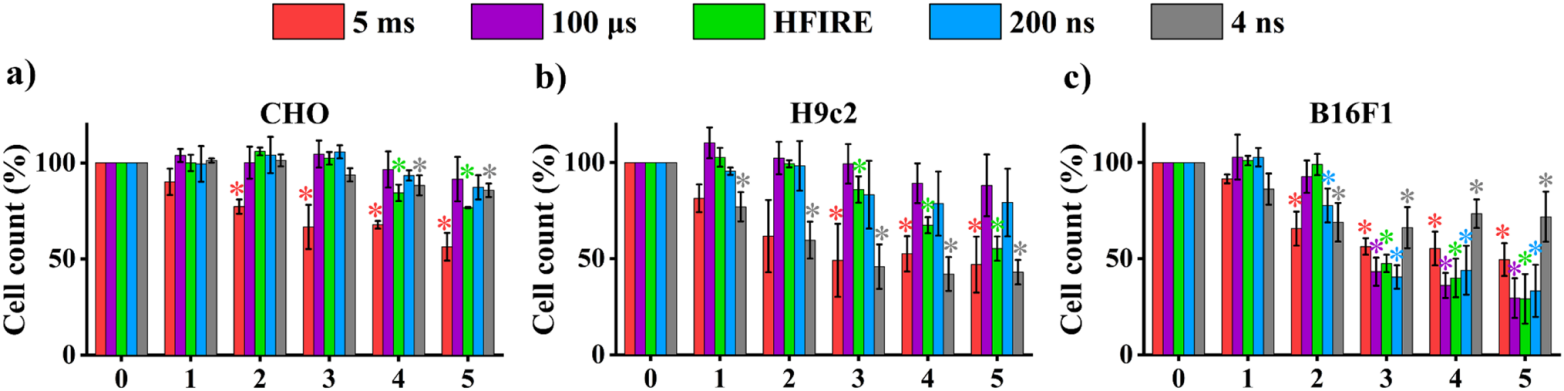
Cell death immediately after pulse treatment. Number of cells obtained at 1 h after electroporation is plotted against the intensities of electroporation (in amplitude for 5 ms, 100 µs, HFIRE, 200 ns and number of pulses for 4 ns). Amplitudes and pulse numbers are described in Table 1. Statistically significant decrease in cell number compared to control is presented. **a** CHO; **b** H9c2; **c** B16F1. 0–Sham control (0 V; 0 pulses) for 5 ms, 100 μs, HFIRE, 200 ns, and 4 ns; 1–100 V for 5 ms, 100 μs, and HFIRE, 1000 V for 200 ns, 1000 pulses for 4 ns; 2–150 V for 5 ms, 200 V for 100 μs and HFIRE, 2000 V for 200 ns, 2000 pulses for 4 ns; 3–175 V for 5 ms, 300 V for 100 μs and HFIRE, 3000 V for 200 ns, 3000 pulses for 4 ns; 4–200 V for 5 ms, 400 V for 100 μs and HFIRE, 4000 V for 200 ns, 4000 pulses for 4 ns; 5–250 V for 5 ms, 500 V for 100 μs and HFIRE, 5000 V for 200 ns, 5000 pulses for 4 ns

Changes in membrane permeability were analyzed on flow cytometer. As cells were collected, we observed that the count of events (i.e., cell count) detected using flow cytometry decreased with increasing time after electroporation as well as with electroporation intensity (Fig. S2, Fig. 3). This indicates that a fraction of cells was associated with a disruption of cell, as they could no longer maintain their plasma integrity. Because cells could no longer be detected with flow as entities, they were considered being disintegrated. This disintegration must have occurred already within 1 h after treatment as the cell count decrease was observed already at 1 h.

**Fig. 4:**
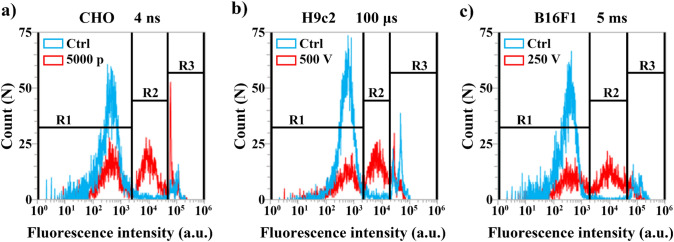
Fluorescence histograms and gate settings. R1 represents live cells, R2 represents cells with increased membrane permeability and R3 dead cells. Blue line is fluorescence intensity profile of sham control and red line is intensity profile of cells exposed to electroporation. Examples of different pulses at 24-h time point after electroporation are presented: **a** CHO; **b** H9c2; **c** B16F1

In interpretation of data obtained with flow, based on sham control, PI fluorescence intensity histogram was first separated on two gates (R1—live cells with autofluorescence/PI negative; and R3—dead cells with very strong PI fluorescence) (Fig. 4). Between the gates of live (R1) and dead cells (R3), additional gate was formed, presenting cells with intermediate PI fluorescence (R2).

In considering R3 (Fig. S1), immediate cell dead (after 1 h) was observed with all electroporation intensity. Lower voltage /pulse number resulted in low cell death count, while high voltage /pulse number resulted in high cell death count. However, with time, number of dead cells decreased, assumingly dead cells were disintegrated and therefore no longer identified by flow cytometer. Furthermore, cells that survived begun to proliferate, so that the ratio between live and dead cells shifted in the direction of the live cells and we could therefore detect only few or no dead cells.

Between the region of live (R1) and dead cells (R3), additional region of intermediate PI fluorescence was identified (R2). This was set do detect cells, with smaller PI intake, which could represent live cell with some damages to the cell membrane (potentially dying cells), similar as it was previously done for distinguishing between permeabilized and dead cells [18] (Fig. 4). If cell death is indeed a dynamic process, this might be reflected in the changes of membrane permeability /membrane damage (R2) at different time points after electroporation. Therefore, to investigate if dynamics of cell death can be reflected in increased permeability of cell membrane, this was analyzed at different time points after electroporation. Results obtained at 1 h after electroporation were considered as immediate cell death, while the results at other time points were considered as delayed cell death.

**Fig. 5:**
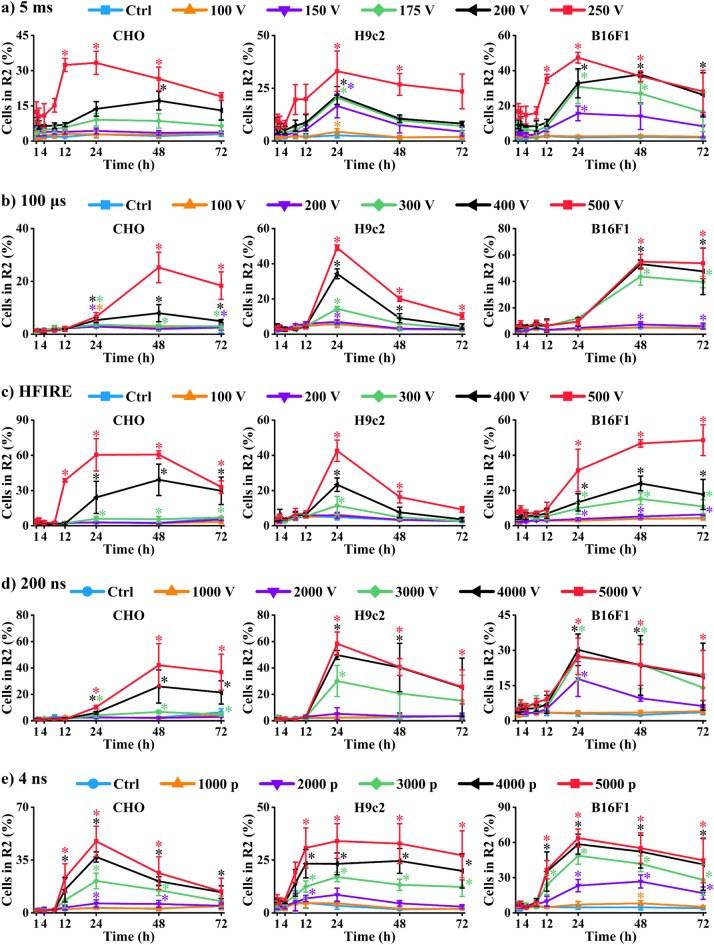
Changes in membrane permeability (R2) detected with PI. Statistically significant differences between PI intake at 1 h after electroporation and Pi intake at different time points after electroporation are shown. **a** 5 ms; **b** 100 µs; **c** HFIRE; **d** 200 ns; **e** 4 ns. For all the pulses left is CHO, middle H9c2, right B16F1

Interestingly, no changes in membrane permeability (R2) were present when pulses with low-intensity electroporation (low voltages or pulse numbers) were used. However, with increasing electroporation intensity, increased membrane permeability was observed in R2 (Fig. 5). The highest increased permeability was caused with the highest electroporation intensities used. This was usually observed at 12- or 24-h time point, depending on cell line and pulse properties. Initial observation of disrupted membrane increased by the next time point. This indicates that higher cell number has disrupted membrane or that initial damage to the cell became more severe, allowing more PI to enter the cell. After reaching the highest signal for disrupted membrane (mostly this was at 24-h time point), the signal decreased. This could happen as a result of repair mechanism activation leading to cell survival (signal shifting to R1) or delayed cell death caused by large levels of stress leading to the final cell death pathways (signal shifting to R3). Furthermore, it is possible that after 24 h, the ratio between live and dead cells shifted to live cells, due to cell proliferation, which decreased the signal of dead cells (signal shifting to R1). Obtained results show that cell death after electroporation is a dynamic process, which is present in all cell lines irrespective of pulse duration or types. However, the results show that dynamics of cell death depends on electroporation intensity.

**Fig. 6:**
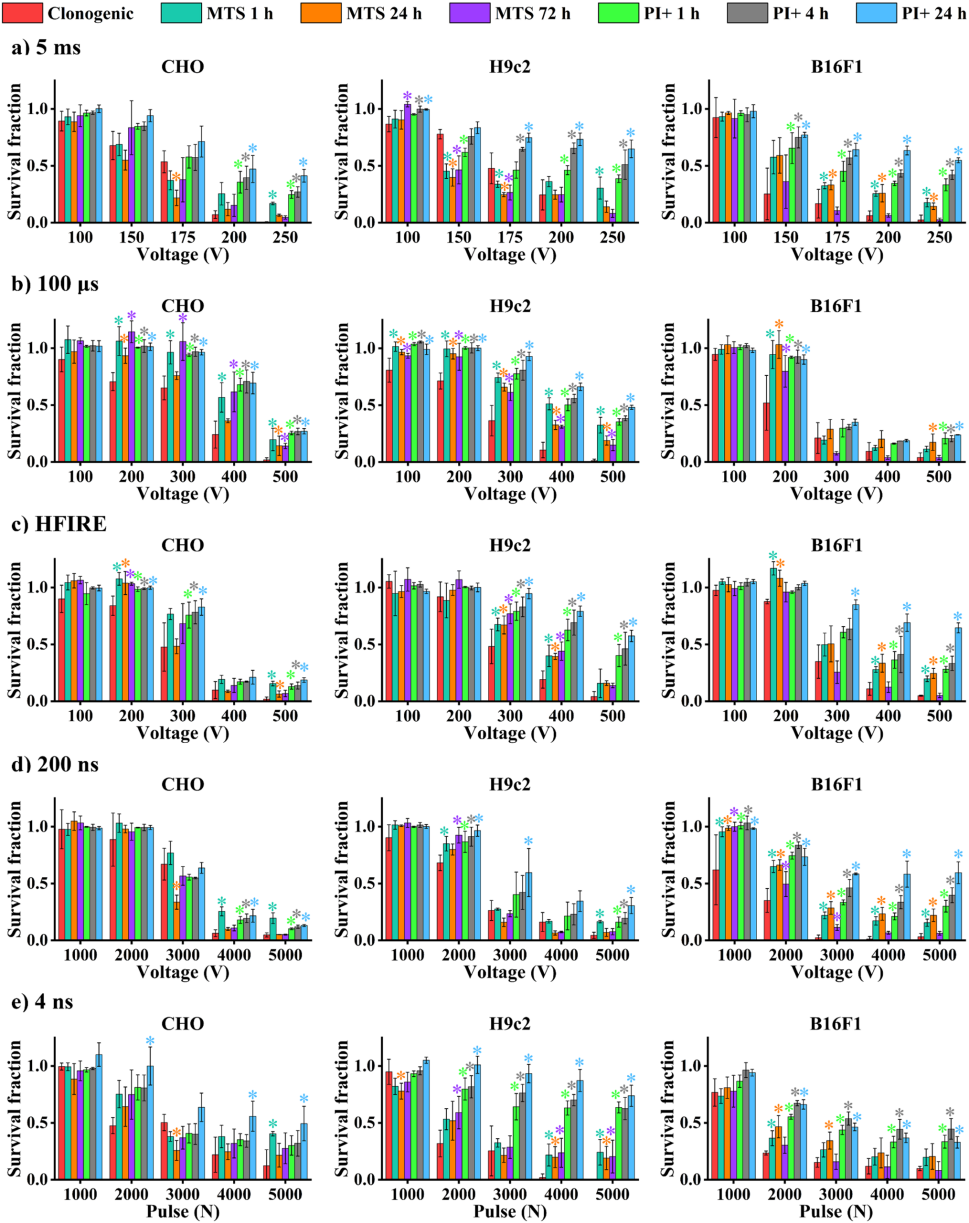
Comparison of different viability assay at various time points after electroporation, based on PI analysis of R2 + R3. Survival of different assays obtained at different times after electroporation is plotted against the intensities of electroporation (in amplitude for 5 ms, 100 µs, HFIRE, 200 ns, and number of pulses for 4 ns). Amplitudes and pulse numbers are described in Table 1. Statistically significant decrease in viability compared to clonogenic assay is presented. **a** CHO; **b** H9c2; **c** B16F1

To assess i) when would be the best time for analysis to assess cell death most accurately and ii) which assay would be the most appropriate to do, we compared the results of different viability assays obtained at different time points. MTS and PI assays performed at different times were compared to results obtained with clonogenic assay, known as the most reliable assay for assessing viability (Fig. 6). Clonogenic assay was compared to MTS obtained at 1, 24, and 72 h after pulse treatment and membrane integrity assay (PI assay) after 1, 4, and 24 h. To compare viability assays as they are usually used, for PI analysis, PI-positive cells (R2 + R3) were taken for analysis. Interestingly, membrane integrity assay performed after 24 h stands out the most, probably due to disintegrated dead cells, which affect the analysis on flow cytometry. Similar behavior was observed for membrane integrity assay performed after 1 h; however, this is more evident when high voltages are used, which makes membrane integrity assay the most unreliable to assess cell death. Interestingly, some results of MTS and clonogenic assay completely overlap, while others vary between 20 and 30%, with MTS exhibiting higher survival than clonogenic assay. Nevertheless, they all exhibit similar trend, which seems to be different for every pulse duration, type, and cell line.

**Fig. 7:**
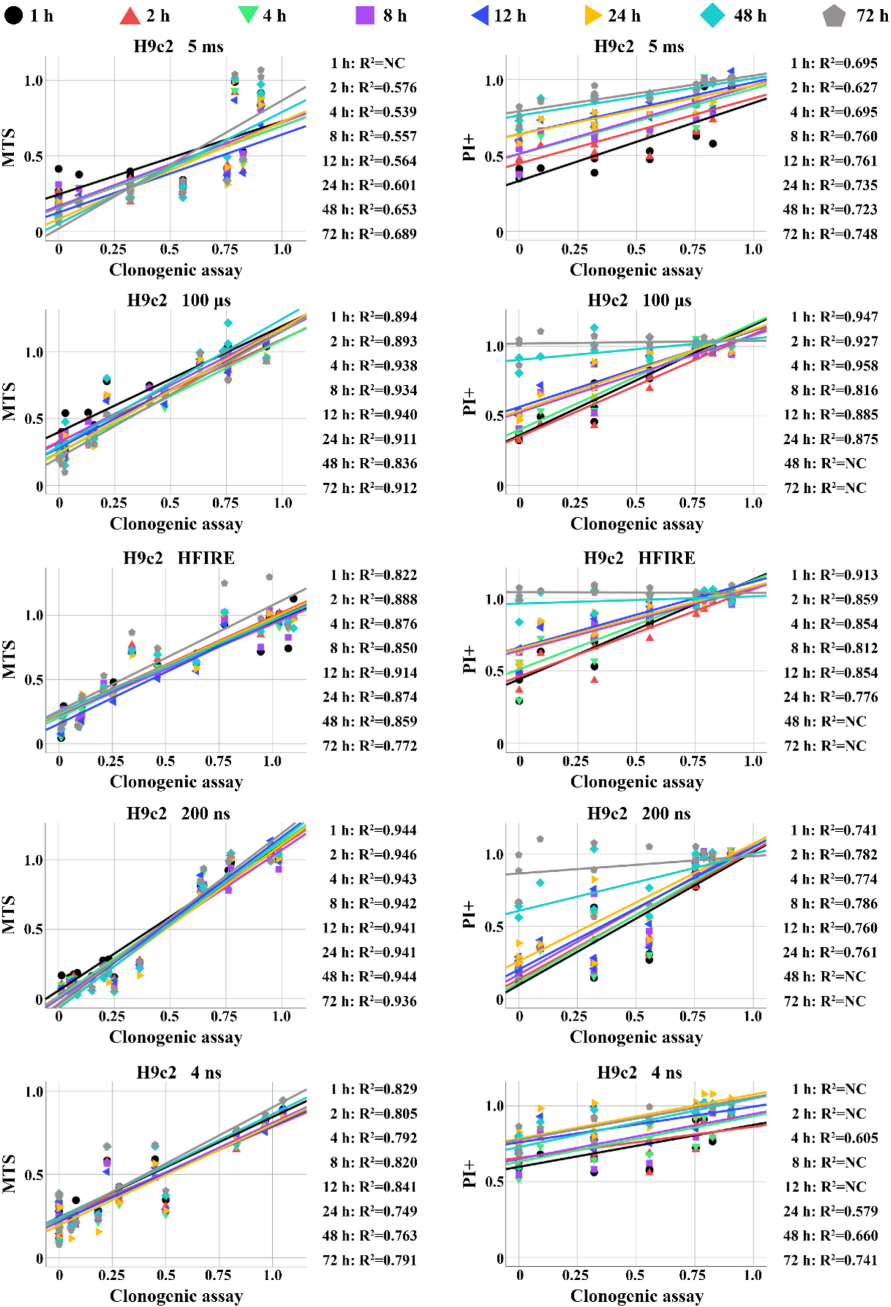
Correlation analysis of clonogenic—MTS assay and clonogenic—PI assay performed on H9c2 cells. NC stands for no correlation, i.e., correlation was below 0.5

Additional correlation analysis based on R2 + R3 (H9c2 is presented in Fig. 7, B16F1 in Fig. S4 and CHO in Fig. S5) showed the strongest correlation between clonogenic assay and PI assay performed between 1 and 2 h after pulse treatment in H9c2, between 1 and 4 h in B16F1 and between 1 and 12 h for CHO. However, in some cell line and pulse treatment, strong correlation was observed even at 24 h and more. Nevertheless, since this was not present consistently, we estimate that in general the best time to use PI in viability assay would be up to 2 h after pulse treatment. The comparison with clonogenic assay was still off, as rarely, we could achieve correlation in the range higher than 0.9 (i.e., strong correlation) between PI and clonogenic assay. MTS compared to clonogenic assay showed stronger correlation than PI assay compared to clonogenic assay.

Because PI signal analysis was separated into different signals for detecting dying (R2) and dead cells (R3), another comparison between viability assays was performed. This time, only dead cells (R3) were taken into analysis (Fig. S6). Signal of PI at 24 h was the only one that strongly differs from previous PI signals in R2 + R3 analysis (Fig. 6). PI signal at 24 h in R3 was much higher than in R2 + R3, making PI at 24 h even less reliable. Interestingly, the analysis of correlation using only R3 (Fig. S7) showed the strongest correlation between clonogenic assay and PI 1–2 h after pulse treatment. Strength of the correlation decreased at 4 and 8 h after treatment. At 12 and more hours after treatment, correlation was mostly non longer existent. This was not observed when R2 + R3 analysis was performed.

## Discussion

Cell death caused by electroporation has been studied since the beginning of electroporation studies. Nevertheless, the mechanisms of cell death and signaling pathways after electroporation are still not well understood [17]. In general, it was considered that apoptosis is the only regulated form of cell death, but recent findings have revealed additional regulated death pathways, including the death of cells that are damaged, infected, or no longer needed in development [4]. Furthermore, cell death can either be a completely independent event having no effect on neighboring cell or affecting neighboring cell viability, either negatively, through what is called a bystander effect, or positively, by providing a survival advantage [25]. So far four types of cell death have been reported in electroporation-based therapies (programed apoptosis, necroptosis, and pyroptosis) and non-programed necrosis [17] as well as bystander effect by subjecting cells that lie near the electroporation region to electroporation effect [29]. The dynamics of the cell populations after electroporation, as a consequence of different death types activation and bystander effect, is being investigated as the dynamics of cell death [13, 16, 33].

In in vivo application of electroporation, both IRE in RE are inevitably present, as cells are exposed to different electroporation intensity. The highest intensity is in the close proximity of the electrodes. From there, intensity decreases with increasing distance from the electrode [3, 36]. Due to different electroporation intensity, different types of cell death resulting in different dynamics of cell death are expected in tissue. Currently, dynamics of cell death was shown using conventional IRE (100 µs), HFIRE, and 300 ns pulses [13, 16, 33]. To further explore the understanding of cell death dynamics, pulses of different durations and polarities were used, starting at nanosecond length and going up to millisecond. With such range of pulse parameters, we assessed dynamics of cell death linked to cell damages, which are caused by nanosecond pulses, bipolar HFIRE pulses, classical monopolar (IRE or ECT) pulses, and millisecond pulses (GET). In addition, this approach allowed us to assess if the dynamics of cell death is specifically related to pulse parameters or represents a more general response of the cell population. Furthermore, using three different cell lines allowed us to investigate (to some extent) if dynamics of cell death is cell type dependent.

We were hoping that consideration of dynamics in cell death could improve the use of less time-consuming viability assay after electroporation. Comparison of different viability assays performed at different time points after electroporation, taking into consideration immediate and delayed cell death, showed variations in viability assessment. Clonogenic assay still seems to be the most accurate to assess IRE. However, MTS performed at 24 h and later is quite close. Why this is not always the case remains unknown. On the contrary, assessment of cell death on flow cytometer through PI internalization proved most unreliable when compared to clonogenic assay. It seems that clonogenic assay is and will be the preferred and most reliable cell death viability assay.

Regardless of the exact values of viability, all cells seem to respond to electroporation in a similar way, with the respect to cell death, as viability decreases with an increase of electroporation intensity as shown by all viability assays. However, dynamics of cell death determined by changes in metabolic activity and changes in membrane integrity may be different in different cell lines. Furthermore, dynamics of cell death, determined by changes in metabolic activity and changes in membrane integrity, was observed in all cell lines when higher electroporation intensities were used (i.e., causing higher than 50% of immediate cell death at 1 h).

Changes in metabolic activity are used to assess direct or indirect damages to mitochondria. Previously, metabolic activity after electroporation with HFIRE pulses was investigated [16]. Increase in electroporation intensity resulted in immediate decrease in metabolic activity. According to our results, the dynamics of metabolic activity is different in different cell lines. In CHO, decrease in metabolic activity was much more evident and similar to previous observations in first 24 h [16]. After 24 h, metabolic activity of CHO cells started to significantly increase up to 72 h, presumingly due to cell proliferation. In H9c2, low decrease in metabolic activity was observed in the first 4 h, however this decrease was statistically insignificant. Furthermore, metabolic activity seemed to be stable for the next 24 h. After 24 h, we expected the increase in metabolic activity due to cell proliferation, however, metabolic activity between 24 and 72 h was not as obvious as in CHO. In addition, even lower metabolic activity was observed, indicating that cell death (based on changes in metabolism) can be delayed up to 72 h. Cell line B16F1 seemed to behave differently from CHO and H9c2, as the metabolic activity increased over the first 24 h and afterward start do decrease. We, however, suspect that these are erroneous results caused by melanin released by B16F1, with an absorbance spectrum at 300–700 nm. As reduction of MTS is detected at 490 nm, absorbance of melanin and reduced MTS may overlap, resulting in overestimation of survival.

Changes in metabolic activity seem to be affected by pulse characteristics, as all pulses did not induce such evident changes in metabolic activity over 72 h. This could also reflect in H9c2 needing more time to restore their proliferative capacity. Interestingly, no changes in metabolic activity were detected after electroporation with 4 ns pulses in CHO and H9c2 cell lines, suggesting that these pulses might induce different damages in the cell. According to obtained results on CHO and H9c2, we can conclude that electroporation may affect metabolic activity depending on the cell line. Interestingly, our results do not confirm H9c2 cells to be more susceptible/sensitive to electroporation than other two cell lines as often assumed [10, 23]. In the broadest sense, H9c2 cells responded quite similar to electroporation as shown for various types of pulses (waveforms) and pulse parameters and as determined by different viability assays.

Internalization of PI has been used in electroporation studies to determine cell death at different time points in electroporation, but which timepoint would give us the best estimation of cell death remains to be elucidated. Dynamics of cell death have been so far confirmed on adherent cells via membrane integrity detection [13, 33]. We investigated membrane integrity on cells in suspension. Membrane integrity was assessed by internalization of PI, as PI fluorescence histogram was separated into three categories of cells: cells that were PI negative (live cells); cell with low PI signal, belonging to live cells with some damage to the membrane, allowing some PI to enter the cell; and strongly PI-positive cells (dead), as the membrane lost its integrity and large amount of PI entered into cells. Obtained results show that high electroporation intensity caused changes in membrane integrity 12–24 h after electroporation, possibly resulting in delayed cell death. Furthermore, this suggests that cell death estimated by PI internalization within few hours (< 12 h) after electroporation may not provide a good estimation of cell death. In addition, this dynamics in cell membrane integrity is present in all cell lines, irrespective to pulse characteristics.

Our results using three different cell lines and broad range of pulse parameters and pulse types also demonstrate that generally, PI assay is the most unreliable at 24 or more hours after pulse application, while PI assay performed at earliest hours after pulse application provides comparable results to MTS or clonogenic assay. MTS and clonogenic tests are showing somewhat similar results with MTS often overestimating cell survival (underestimating cell kill) as others have already observed before [7, 35]. Overall considering the dynamics of cell death and underestimation of cell death making clonogenic assay the preferred viability assay. Results of our study also suggest that dynamics of cell death is only present when high-intensity electroporation is used. However, dynamics of membrane integrity shows that dynamics of cell death is observed in all cell lines irrespective of pulse characteristic. This was in part confirmed also with metabolic assay. Additional research into cell death need be performed to further investigate the cell death pathways.

### Supplementary Information

Below is the link to the electronic supplementary material.

Supplementary file1 (PDF 2279 kb)
