# Supplementary material for: Dynamics of Cell Death Due to Electroporation Using Different Pulse Parameters as Revealed by Different Viability Assays
Source: Ann Biomed Eng. 2023 Sep 13;52(1):22–35. doi: 10.1007/s10439-023-03309-8 (PMC10761553; doi:10.1007/s10439-023-03309-8)
Supplement: Supplementary file 1 — Supplementary file1 (PDF 2279 kb) [file 10439_2023_3309_MOESM1_ESM.pdf]

Dynamics of cell death due to electroporation using different pulse parameters as revealed by different viability assays

Supplementary material

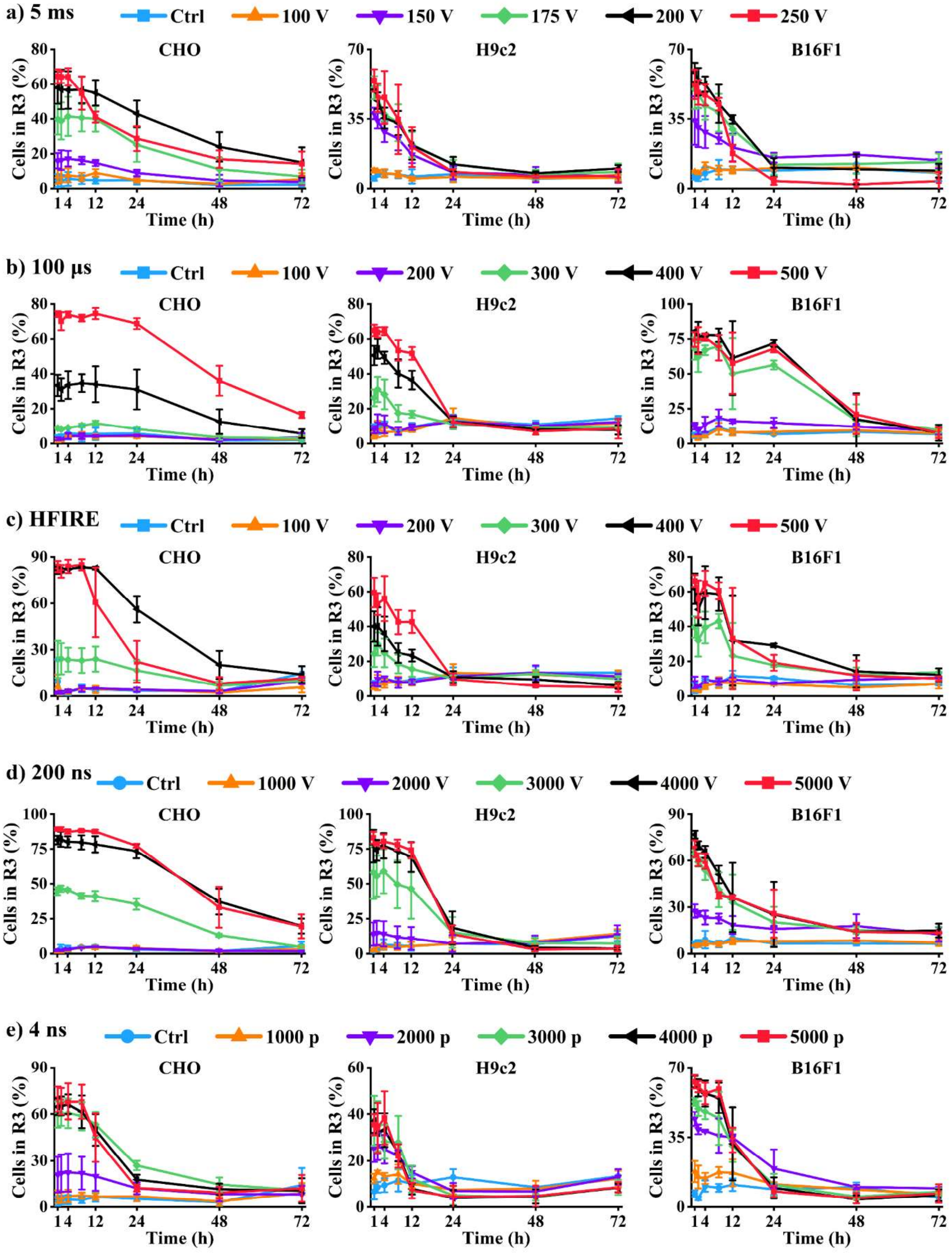

Figure S1: Dead cells (R3) detected with PI over 72 hours. a) 5 ms; b) 100 µs; c) HFIRE; d) 200 ns; e) 4 ns. For all the pulses left is CHO, middle H9c2, right B16F1.

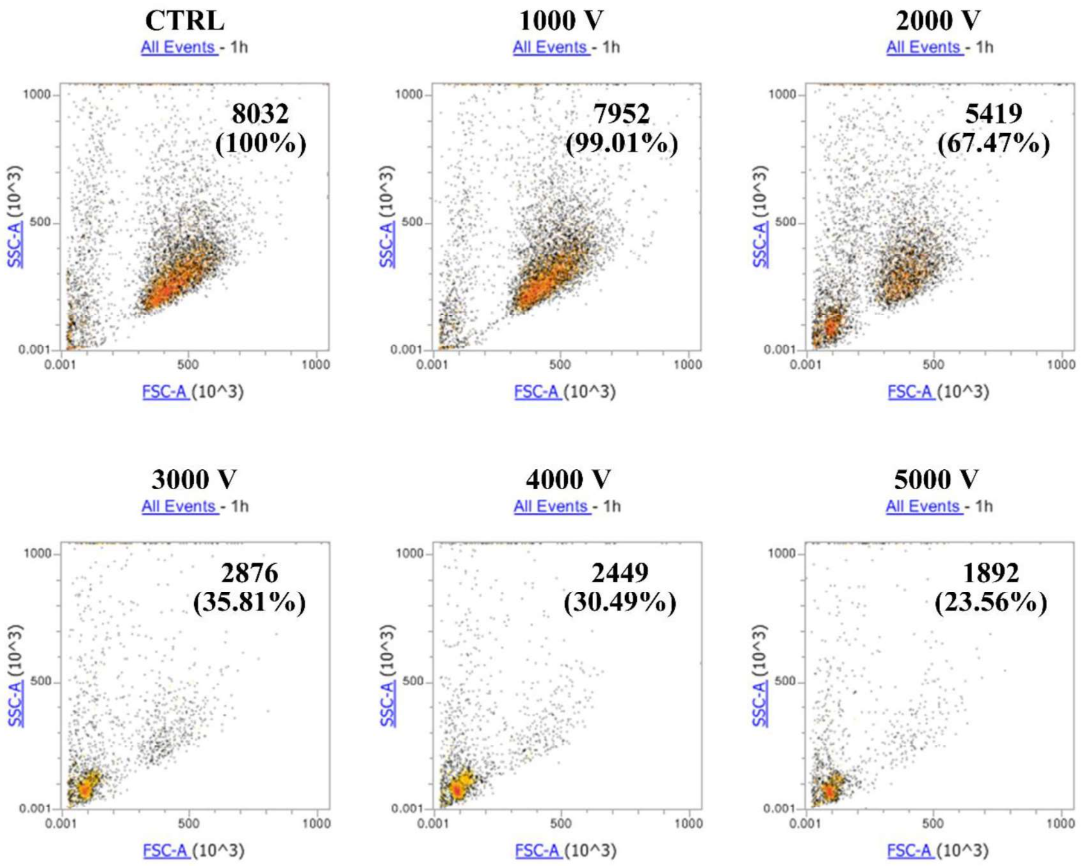

Figure S2: Cell count one hour after electroporation. An example is presented for 200 ns pulses on B16F1; however, all pulse treatments and all cell lines had the same trend. With the increase of electric field, the number of cells detected with flow cytometer decreases.

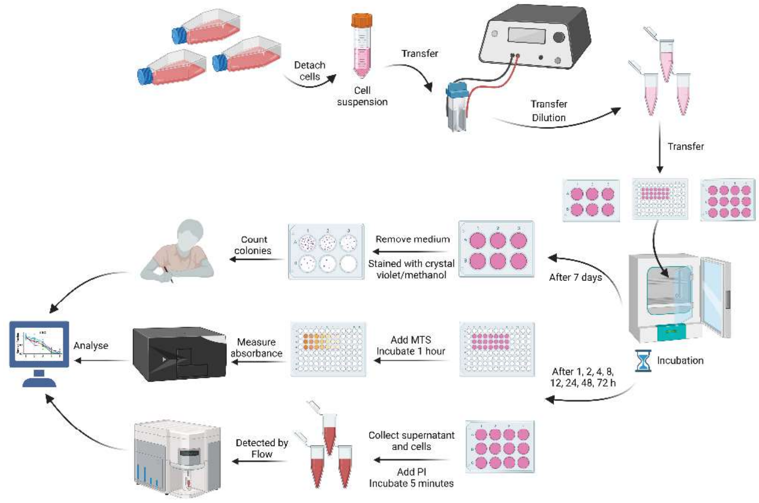

Figure S3: Schematics of the experiment

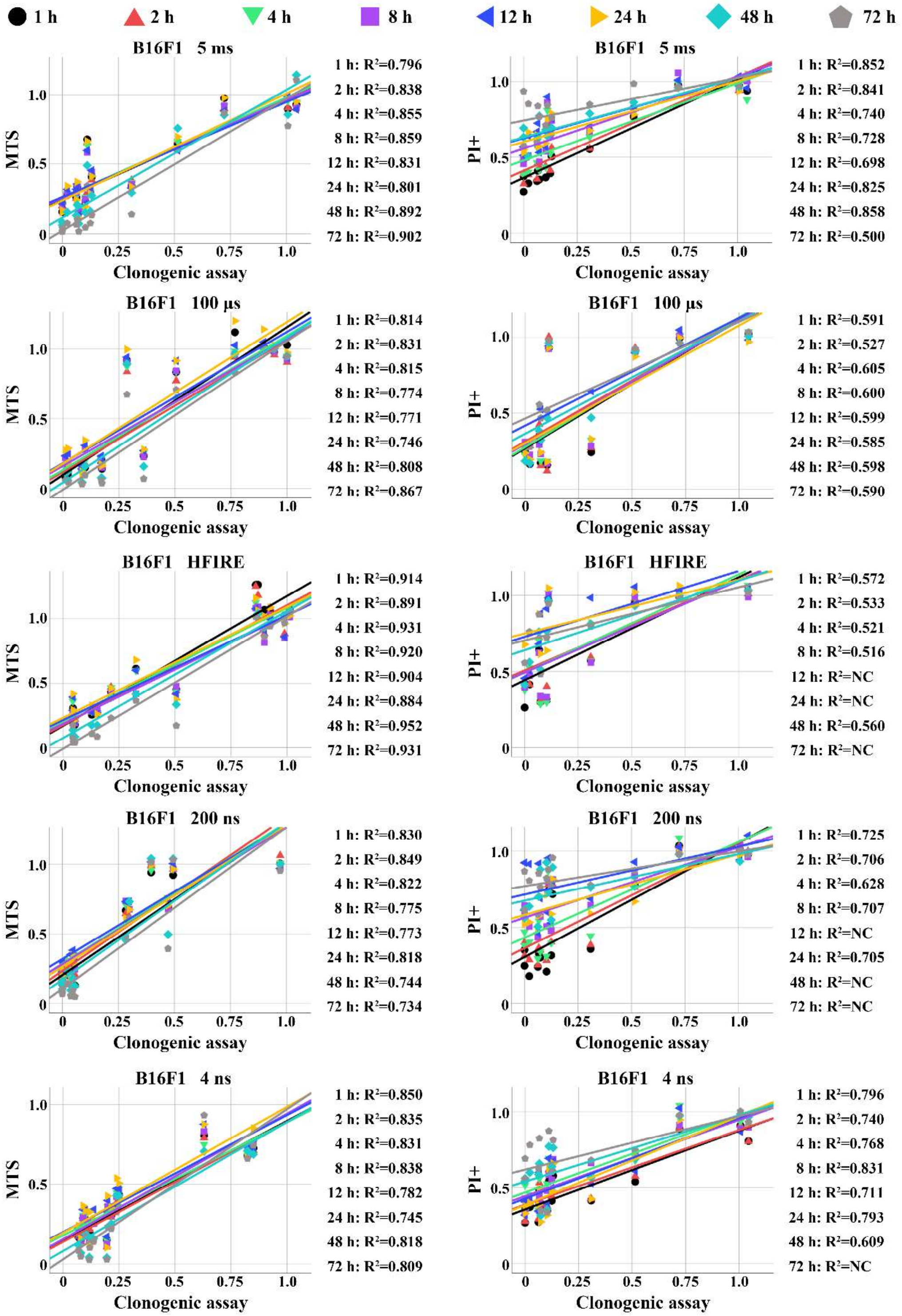

Figure S4: Correlation analysis of clonogenic - MTS assay and clonogenic - PI assay performed on B16F1 cells. NC stands for no correlation, i.e. correlation was below 0.5.

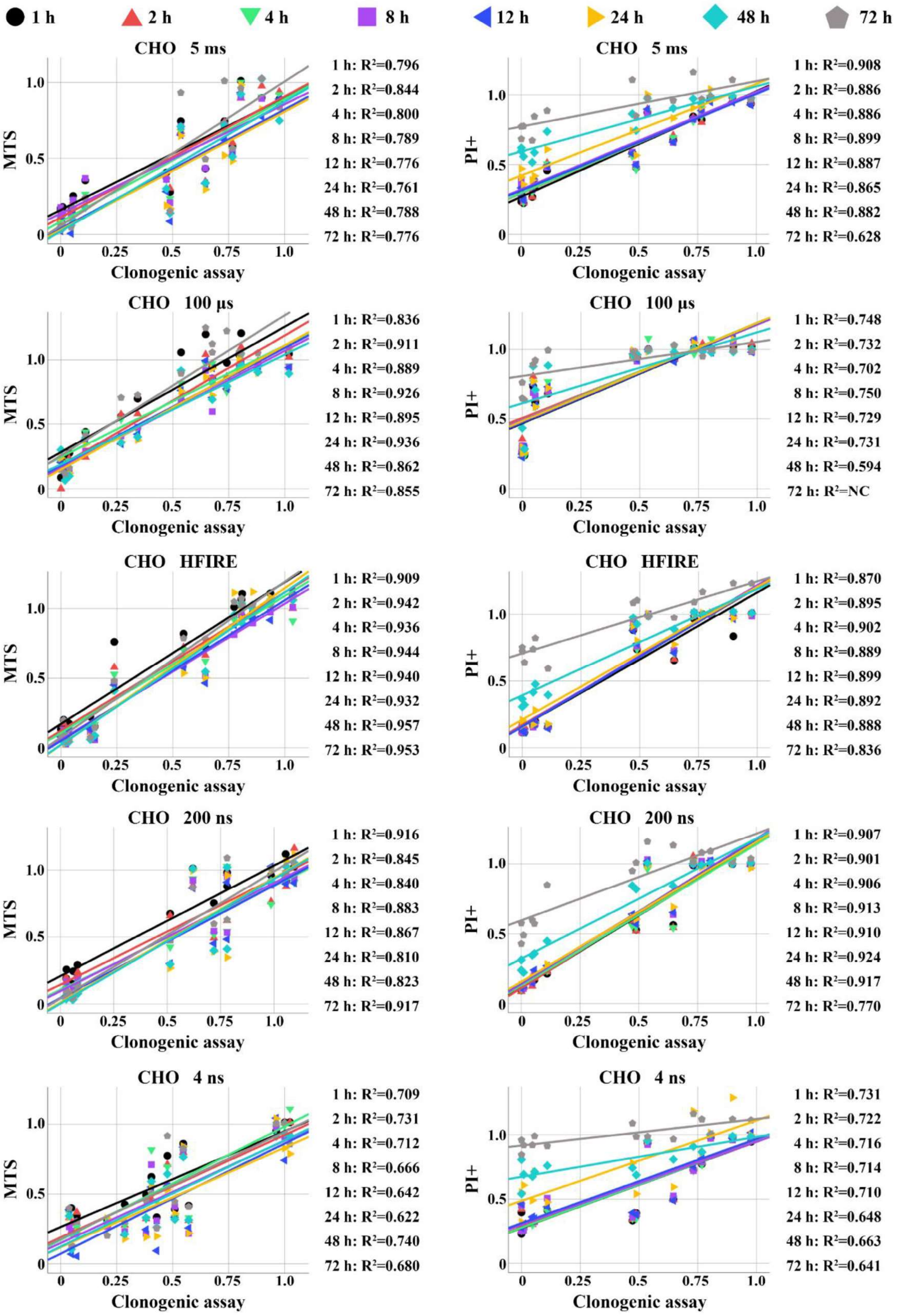

Figure S5: Correlation analysis of clonogenic - MTS assay and clonogenic - PI assay performed on CHO cells. NC stands for no correlation, i.e. correlation was below 0.5.

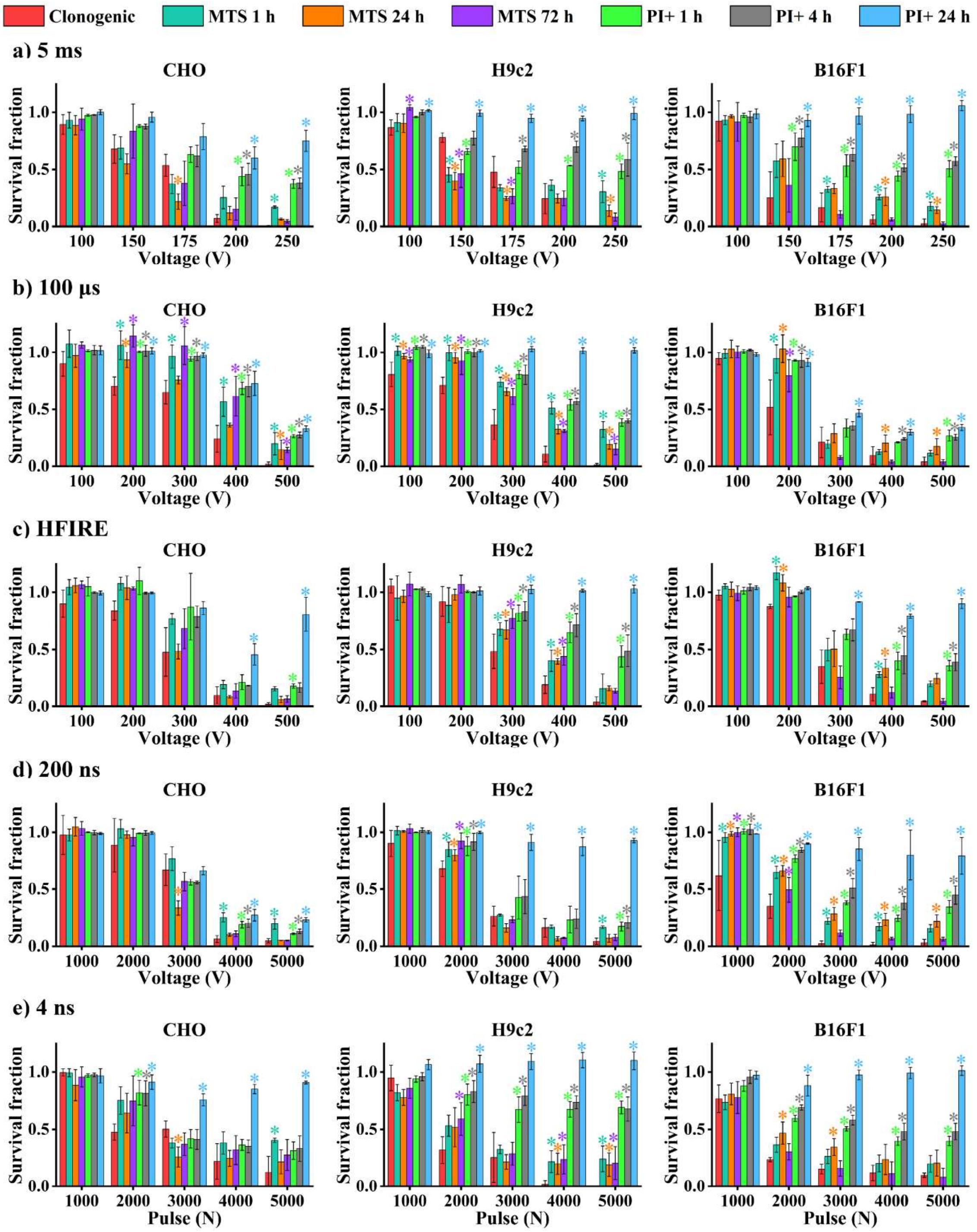

Figure S6: Comparison of different viability assay at various time points after electroporation, based on PI analysis of only R3. Survival of different assays obtained at different times after electroporation is plotted against the intensities of electroporation (in amplitude for 5 ms, 100 µs, HFIRE, 200 ns and number of pulses for 4 ns). Amplitudes and pulse numbers are described in Table 1. Statistically significant decrease in viability compared to clonogenic assay are presented. a) CHO; b) H9c2; c) B16F1

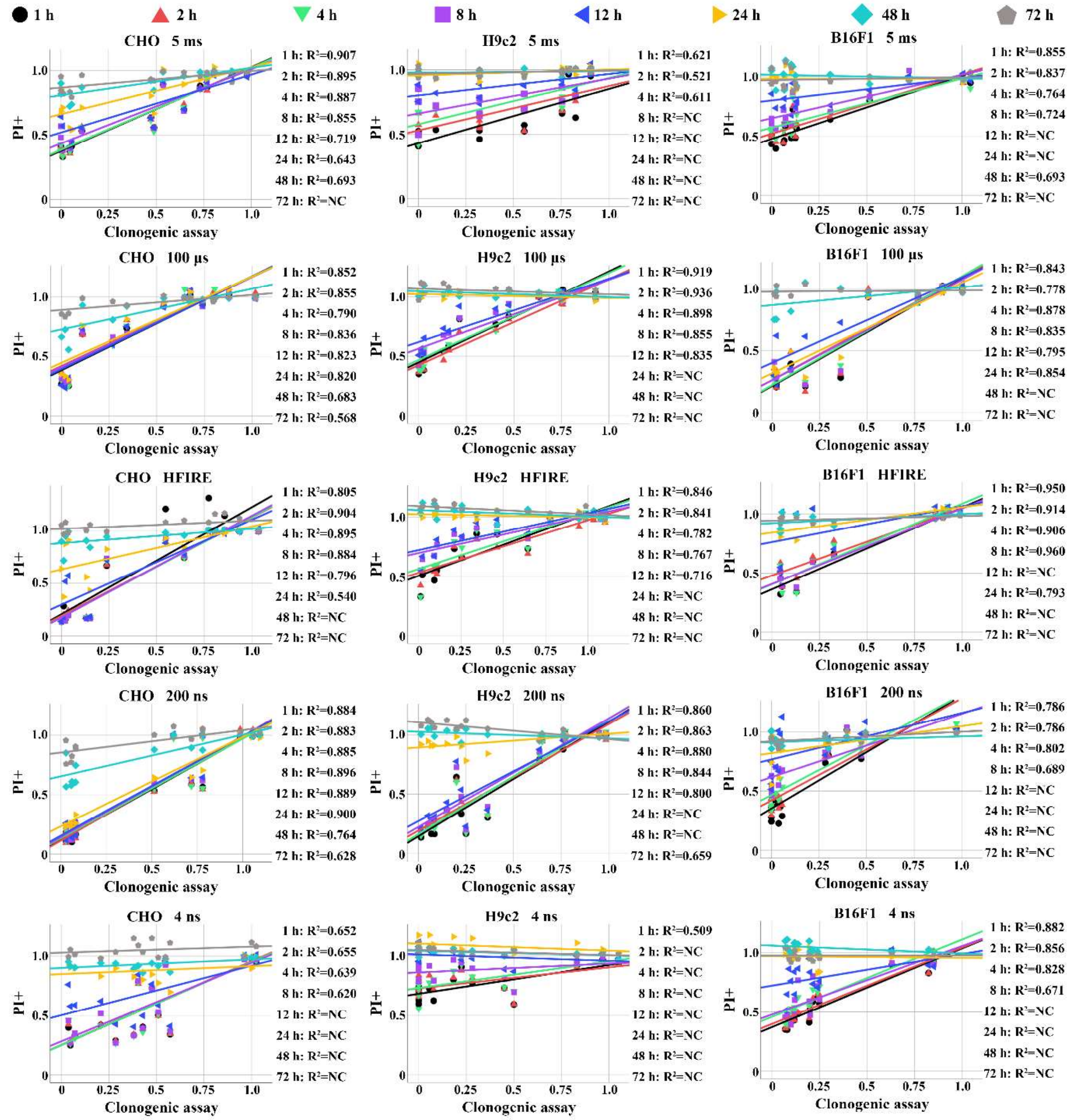

Figure S7: Correlation analysis of clonogenic - MTS assay and clonogenic - PI assay performed on CHO, B16F1 and H9c2 cells based on R2. NC stands for no correlation, i.e. correlation was below 0.5.
